# Methods for using worker-centered research to improve food donation and reduce wasted food in a grocery retail setting

**DOI:** 10.3389/fpubh.2025.1609717

**Published:** 2025-07-18

**Authors:** Kaitlyn Harper, Steffanie Espat, Lee Davis, Nicole Labruto, Roni A. Neff

**Affiliations:** ^1^Department of Environmental Health and Engineering, Johns Hopkins University Bloomberg School of Public Health, Baltimore, MD, United States; ^2^Center for Creative Impact, Maryland Institute College of Art, Baltimore, MD, United States; ^3^Department of Anthropology, Johns Hopkins University Krieger School of Arts and Sciences, Baltimore, MD, United States

**Keywords:** human-centered design, participatory research, worker-centered approach, qualitative methods, ethnography, food donation, wasted food, grocery retail

## Abstract

**Introduction:**

This project took a novel approach to reducing wasted food and improving food donation by prioritizing and centering the ideas and experiences of frontline grocery retail workers, who were integrally involved in each step of the research process. In this paper, we describe in detail the methods used in the Food Donation Champions Project, a worker-centered project in collaboration with a large US grocery retail chain. We provide the context, process, and lessons learned through our partnership with corporate leaders and frontline workers.

**Methods:**

This project was conducted using a convergent, human-centered design process, involving design, public health, and anthropology research methodologies. The process involved six steps: planning, research, synthesis, ideation, prototype development and testing, and strategy finalization. We collected qualitative data through interviews and observations with grocery retail workers, members of corporate leadership, and stores' donation partners (i.e., food pantries and food banks). Frontline workers informed this research strategy and participated in all stages of analysis and strategy development.

**Discussion:**

The process and findings described in this paper provide researchers and leaders in grocery retail a guide to a novel methodology and research approach that may be used to enhance projects that elevate the lived experience of people most central to addressing social and environmental problems.

## 1 Introduction

### 1.1 The complexities of wasted food

**Figure 1:**
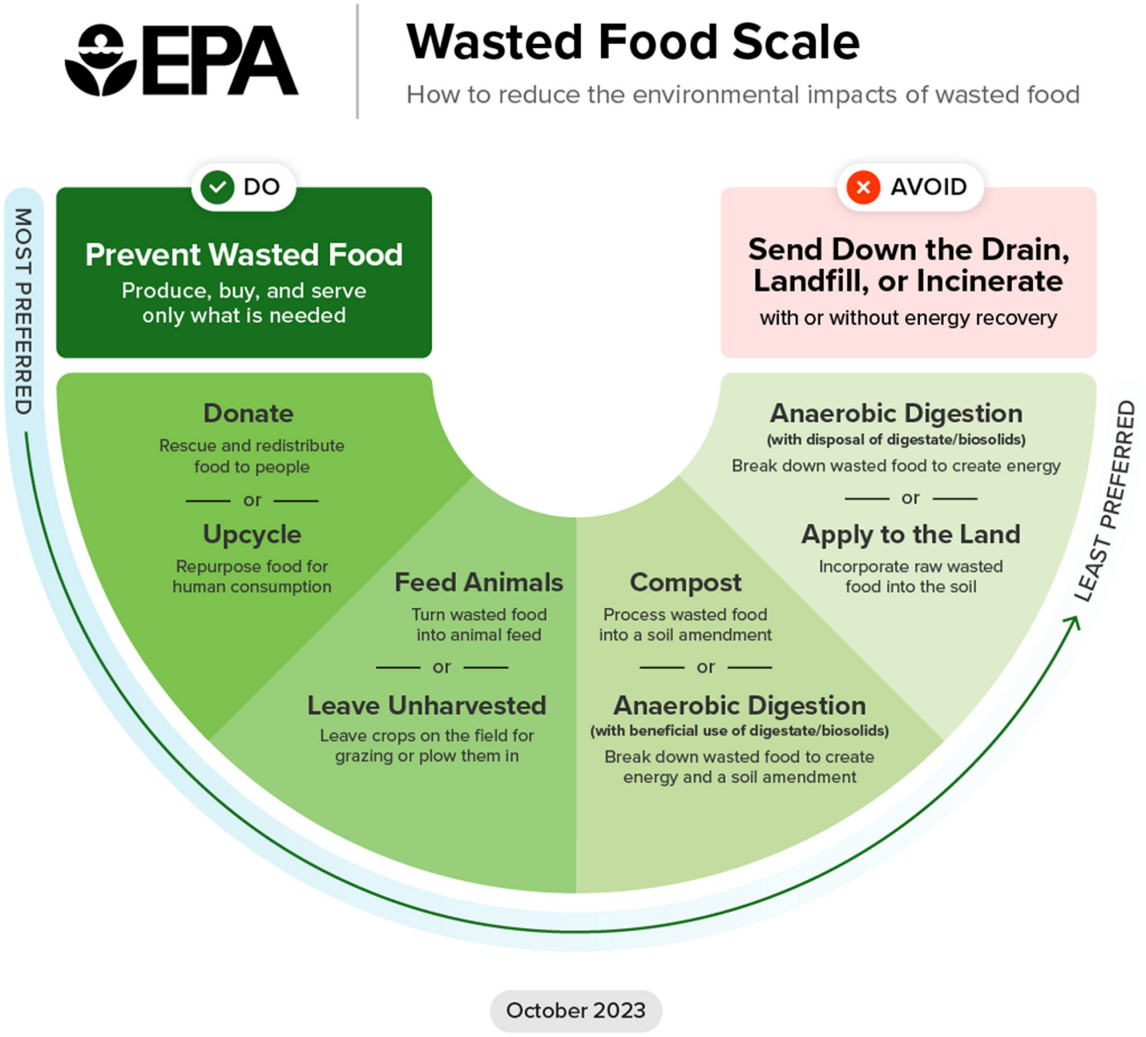
US Environmental Protection Agency Wasted Food Scale.

In the United States (US), more food is wasted per person than in almost any other country in the world (1). Approximately 40% of the 235 million tons of food produced by the US food system each year goes unsold or uneaten. In 2023, the US Environmental Protection Agency created the Wasted Food Scale (Figure 1), which describes a range of preferred destinations for surplus food (2). The scale first prioritizes feeding people through food donation, then feeding animals. When these preferred destinations cannot be met, the scale indicates that wasted food should be left unharvested in fields, composted, or anaerobically digested. The least desired destinations for food are landfills, waste incinerators, and wastewater systems (i.e., through garbage disposals). Currently, wasted food most often ends up in these least desired locations (3).

Wasted food is considered a “wicked problem”, defined as an extremely complex issue that lacks a definitive solution and requires systems-level strategies to address many nuanced and interconnected components (4). Indeed, wasted food generated at each step of the food system is influenced by a wide range of factors including economics, politics, public health, infrastructure, culture, and human behavior (5). Researchers from numerous disciplines focus on these various aspects, but research is often siloed, and a convergent approach is needed to address the complexity and nuances of the problem. Convergent research “brings together intellectually diverse researchers and stakeholders to frame the research questions, adopt common frameworks for addressing them, and create and implement innovative scientific approaches for their solution” (6, 7). Convergent research differs from other multidisciplinary methods by combining epistemologies and approaches from multiple disciplines to develop new methods, research tools, and ways of thinking and communicating that otherwise may not have been conceived. Successful convergent research on wasted food brings together researchers in diverse areas and integrates their approaches to better understand why, when, and how wasted food occurs, and creates meaningful and long-lasting solutions.

### 1.2 Wasted food in grocery retail

Approximately 4.5 million tons, or 5% of all wasted food, is generated by the grocery retail sector (8). In 2023, 17% was turned into animal feed and 18% was composted, while nearly 35% of wasted food in retail ended up in the landfill or incinerated. Notably, only 19% of retail wasted food was donated to people in need (9). A growing body of literature describes the problem of wasted food in retail settings. In their comprehensive review, De Moraes et al. (10) identified numerous causes of retail food waste in the supply chain and operations management and sorted them into six categories: method (e.g., lack of formal procedures to regularly rotate shelves); measurement (e.g., inadequate demand forecasting, excess production); material (e.g., damaged packaging, confusion over date labels); machine (e.g., cold storage breaking, lack of refrigerated transport); people (e.g., incorrect handling of delicate items like produce, lack of training); and environment (e.g., problems with seasonality, strict food safety laws) (10). Numerous studies have proposed solutions to reduce wasted food in retail settings (10–15), but only a few of these have included frontline workers' perspectives (11, 14, 15). Reducing wasted food in retail settings depends on frontline workers and their ability to implement anti-waste activities on the ground. However, frontline workers are not always included in the creation of store procedures, guidelines, or efforts to enact change.

### 1.3 Worker-engaged strategies for reducing wasted food

Recently, a few case studies have has tested workers' ideas for reducing wasted food in production and manufacturing. The Pacific Coast Food Waste Commitment (PCFWC), a group of food businesses publicly committed to private sector action to reduce food waste (16), and TripleWin Advisory, a consultancy firm specializing in sustainability and food loss reduction (17), partnered with three companies to create and pilot test the effect of worker-designed interventions to reduce wasted food. In their first project at Bob's Red Mill, a grain milling and packaging facility, a worker-designed intervention resulted in nearly a 70% reduction of wasted food on the manufacturing line (18). At Land O'Lakes, workers designed a strategy that reduced waste of a particular food product by 74% (19). Most recently, workers at Fresh Del Monte, a produce producer and distributor, designed an intervention that recovered 53% of a product that would have otherwise been wasted (20). In another collaboration between PCFWC and Stanford Food Institute, foodservice staff generated over 120 strategies to reduce waste in university dining halls (21). The impact of these efforts displayed the impact and importance of engaging workers in creating solutions. However, none of these projects have focused on grocery retail settings. Given the vast experience and expertise of frontline workers and the priority on addressing wasted food in retail, further research is needed to understand implementation and impact of strategies designed by workers in grocery retail settings.

### 1.4 Project goals and scope of this paper

In this paper, we describe the methods of the Food Donation Champions Project, a study conducted in partnership with “Company X”, a large US grocery retail company (Note: the outcomes of this study will be presented in a forthcoming paper). Company X uses numerous food waste reduction tactics and diverts over 300 million pounds of inedible food to compost and anaerobic digestion and donates between 70 and 100 million pounds of edible food to local donation partners each year. Company X uses a company-wide donation program that aims to redirect edible food that might otherwise be discarded to donation. This program was updated after the end of the COVID-19 pandemic. However, even after the updates, some stores continue to struggle to donate food regularly.

Through the Food Donation Champions Project, researchers from Johns Hopkins University (JHU) and Maryland Institute College of Art (MICA) (henceforth, the research team, or researchers) collaborated with Company X corporate leadership and store-level employees to explore and address this challenge. We convened ten frontline workers (henceforth, Food Donation Champions, or Champions) from four Company X stores along the Eastern Seaboard area to achieve three overarching goals: (1) improve the existing food donation program at Company X grocery retail stores; (2) explore the use of a worker-centered model within a limited number of Eastern Seaboard grocery retail stores; and (3) challenge power imbalances that often occur in retail corporations resulting from top-down decision making.

The Food Donation Champions Project asked the novel question: how might unlocking the expertise, creativity, and motivation of grocery retail workers transform food donation? In this study, strategies were generated *by and with* frontline workers to improve food donation and reduce wasted food. This paper provides a case study utilizing an innovative convergent methodology and research approach, which combines elements of anthropology, public health, and design research. We describe the methods used in the Food Donation Champions Project, as well as the context, process, and lessons learned through our partnership with Company X corporate leaders and frontline workers.

## 2 Methods

### 2.1 Context

Company X operates stores in over half of US states. Stores are grouped by regions, which may include multiple states, such as the company's Eastern Seaboard region. Within each store, there are seven or eight food departments, each led by a Department Manager: Produce, Bakery, Deli, Meat, Seafood, Dairy, Center Store and, in some stores, Coffee (typically operating separately from a kiosk).

Typically, company-wide initiatives—including initiatives related to wasted or unsold food—are communicated from national corporate leadership to regional leadership, including the region's President and a designated corporate representative. Regional leaders typically either communicate directly with relevant workers in their region's stores or relay information to subregional leaders, who communicate directly with workers. Almost all communication happens through regional or subregional virtual calls and/or email.

After the COVID-19 pandemic, the company began implementing an updated version of their food donation program across all stores. At the time, each region's leadership held a 1-hour virtual meeting with store department managers to provide updated guidance about the program and emailed updated donation guidelines. For the subsequent 6 months, corporate leadership tracked the amount of food donated from each store and each department. Although some stores in some regions began donating consistently, uptake of the program was inconsistent. The Eastern Seaboard was among the regions with the lowest uptake. Thus, it was an ideal location to explore store-level barriers and strategies to improve the food donation program.

### 2.2 Convergent approach

The Food Donation Champions Project was conducted using a worker-centered, convergent process combining research methods from design, public health, and anthropology. Throughout the project, we consciously combined methods from three qualitative research approaches: Human-Centered Design (HCD), Community-Based Participatory Research (CBPR), and Participant-Observation-Based Ethnography (henceforth, ethnography).

HCD is a collaborative, creative process dedicated to understanding the experiences, behaviors, and needs of people at the heart of an issue or problem and designing interventions that better serve their needs and/or alleviate challenges they are facing (22). Although HCD can be used by for-profit corporations to design better or more profitable products, the researchers in this project utilize the HCD process to address social and environmental issues and design interventions to alleviate them. HCD views problems through the perspective of the people directly impacted by a problem or issue—in this case the Champions—and positions their lived experience as expertise. The HCD process has a prescribed set of steps (see Section 2.4) and may also be referred to as “design research.”

CBPR refers to an epistemological and methodological approach in which community members collaborate equally with professionally trained researchers to conduct research activities, such as developing the study questions, designing the methodology, collecting data, and contributing to and disseminating the study findings (23). CBPR and HCD have many similarities, including that they rely primarily (but not always) on qualitative data and the research team and participants both contribute directly to the design and development of the final outcome or product. In both approaches, participants may be, but are not always, included in every step of the research process. Unlike HCD, CBPR does not prescribe specific steps or methods and can include descriptive projects in addition to intervention-focused ones.

Ethnography, a key method used by anthropologists and sociologists, studies the beliefs, social interactions, and behaviors of individuals or groups (24). This requires the qualitative research strategy of participant observation, in which a researcher embeds themself among a group of people in order to gain as complete an understanding as possible of practices, meanings, and structures within the group (25). Ethnographers take detailed written and visual notes and later analyze them. Similar to HCD and CBPR, ethnography prioritizes perspectives and cultural understandings of community members. However, the central focus of ethnography is in gaining knowledge about a community without necessarily taking action, while CBPR and HCD specifically prioritize engaging community members in the research process. Further, ethnography is descriptive while HCD (and often CBPR) focuses on creating and/or implementing interventions.

The research team met regularly throughout the study to plan and revise the research plan, analyze results, and discuss and resolve challenges. Convergence was an important mindset and objective of the project, as a means of evolving new ways of thinking and doing and was intentionally discussed at nearly every team meeting. Although the underlying structure of the research process was based on the steps of HCD (see Section 2.4), aspects of anthropology and public health were infused throughout. In this paper we highlight key moments where our distinct disciplinary approaches converged to develop new ideas and methods of inquiry.

### 2.3 Researcher positionalities

Researchers' positionalities affect the research process, and we practiced reflexivity in every step of the study, from project formation to disseminating results. Reflexivity is a process of self-reflection for researchers to recognize and understand their influence in shaping interactions with participants and the study environment (26). Here, we provide transparency about our process by disclosing information about ourselves that is pertinent to the content of this project, its data, and our conclusions. The authors of this paper identify as white, middle- to upper-middle class and all have advanced academic degrees. None of the authors have extensive experience working in grocery retail, but one (NL) has experience working in the restaurant industry, which shares some similarities to food retail. No authors have experience working in large corporations, but one (LD) has experience collaborating with companies as philanthropic partners. Two authors (KH, RN) have significant experience and knowledge of food donation partners (e.g., food banks and pantries), and all five have expertise in wasted food and/or food systems. Additionally, all five have expertise in community-based, equity-centered research.

### 2.4 Overview

**Figure 2:**
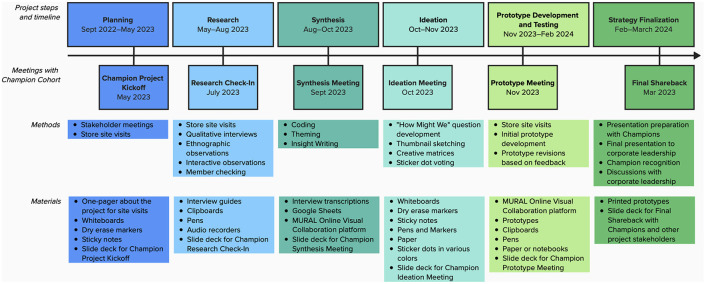
Timeline and overview of the Food Donation Champions Project.

The project was conducted in two phases—“Exploration and Learning” and “Creating and Testing” (*italics* and Figure 2). Phase 1: Exploration and Learning included collaborative *planning* between the research team and members of Company X corporate leadership, recruitment of participants, in-store and interview-based *research*, and data *synthesis*. Phase 2: Creating and Testing included *ideation*, a guided brainstorming process in which the Champions generated ideas for potential food donation-related interventions based on their experiences and the earlier research; *prototype development*, in which the research team and the Champions selected a few ideas, developed models of each idea, and tested their desirability, usability, and feasibility in the four study stores; and *strategy finalization*, in which we integrated feedback gathered during prototype testing and evolved the prototypes into more detailed strategies to share with Company X corporate leadership. Throughout the process, we conducted six in-person meetings with the Champions, corresponding with each of the six steps of research.

### 2.5 Phase 1: exploring and learning

#### 2.5.1 Planning

This project was conceptualized by one member of the research team (RN) and one member of Company X corporate leadership, who were both invested in exploring worker-centered strategies to reduce wasted food. Between September 2022 and May 2023, the research team worked with Company X corporate leadership to develop goals and the scope of work for this project. We met virtually with Company X one to two times monthly and one time in person during the planning period and created and signed three partnership documents: a Memorandum of Understanding, which outlined each parties' intentions for the project; a Non-Disclosure Agreement, which stated that the researchers would not share sensitive data without Company X leadership consent; and an Operations Agreement, which described the differences in roles and responsibilities between the research team and Company X leadership.

The collaboration with Company X presented a unique research opportunity for a variety of reasons. First, it possessed significant potential for affecting change and addressing food waste within one of the largest corporate retailers in the country and within one of the industries with the greatest opportunity for food donation. Second, it provided direct access to grocery retail stores and frontline workers that would otherwise be unavailable for researchers. And third, Company X was, from the beginning, committed to sharing the ideas and lessons that emerged from the research, presenting the unique opportunity of modeling supermarket waste reduction approaches that might be adopted by the retail industry (and others) more widely.

#### 2.5.2 Recruitment

Company X selected four stores in the Eastern Seaboard region to participate in this study. Selected stores represented various geographies (urban, rural, suburban) and sales volumes, a metric used to describe the amount of food and other items sold by stores (i.e., high volume stores sell more than low volume stores). Additionally, the four stores were selected because they were either not donating or donating very little on a regular basis prior to the beginning of the study.

Throughout the study, we collected data from four groups of participants: (1) The Food Donation Champions; (2) Non-Champion workers and Store Directors in the selected stores; (3) members of Company X national- and regional-level corporate leadership whose main jobs or components of their jobs focused on food waste reduction; and (4) donation partners—the food banks and food pantries that picked up and distributed donated food from the four study stores.

To recruit Champions, the research team and members of Company X corporate leadership visited each of the four study stores to begin building relationships with workers and Store Directors, and to promote the study. During these visits, we presented the project verbally and handed out fliers with more information about the study goals and participant expectations. After the initial store visits, each Store Director selected two to four Champions to participate in the study. Individuals were eligible for selection if they were over 18 years old and worked as Department Managers in one of the fresh food departments (i.e., Meat, Seafood, Deli, Bakery, Produce, Dairy, or Coffee) at one of the four participating stores. Department Managers conduct and oversee daily operations of their respective departments, including ordering products, monitoring loss of products (e.g., from damage, waste, or theft) called “shrink”, and providing guidance and support to other workers. Although they are in a leadership role within their department, they also perform tasks on the floor alongside other workers daily (e.g., stocking shelves, cleaning, preparing food) and are therefore considered frontline workers. In this study, the Champions had a range of grocery retail experience, ranging from 2 years to over 30 years, and eight of the ten Champions had over 15 years of experience.

Employees who were interviewed but were not Champions included the Store Director and all Department Managers, Assistant Department Managers, and Inventory Control Clerks (or “Receivers”, who are in charge of food entering and leaving through the back of the store) in each store. To recruit non-Champion members of the store staff, the research team worked with Store Directors and Champions to arrange times to visit the stores during regular work hours. These staff were informed ahead of time that the research team would be visiting and that they might be invited to speak with the research team. During the visits, the Store Director or the Champions introduced the research team to the workers, one-on-one. Workers could decline participation ahead of the store visit or at the time of the store visit. The four Store Directors had already committed to the study, and the research team emailed or called them ahead of the store visit to request interviews.

Four members of Company X national- and regional-level corporate leadership were key partners throughout the study and were also interviewed. Additionally, the Champions recommended interviewing their subregional-level leadership that oversee specific departments across multiple stores. To recruit donation partners, each store provided a list with contact information of the organization(s) that pick up donated food one or more times per week.

Study procedures were determined exempt by Johns Hopkins Bloomberg School of Public Health Institutional Review Board. Oral consent was obtained during the first meeting with the Champions and at the beginning of individual interviews with each participant. Ongoing consent was obtained from the Champions each time they participated in individual interviews or data collection throughout the study.

#### 2.5.3 Data collection

During Phase 1, data collection occurred at the four study stores, Champion meetings, and donation partner sites.

We conducted in-store research between June and August 2023, and members of the research team visited each study store five times during this period. This process was largely rooted in ethnographic methods (25). Prior to beginning visits, the research team's anthropologist (NL) led a discussion with the research team about best practices for participant observation (27). Additionally, the researchers discussed how qualitative interviews are typically conducted in their respective disciplines. We found that the methods were similar across disciplines, but sometimes used distinct vocabulary (e.g., “interview guide” vs. “interview schedule”). We created a convergent standard operating procedure for data collection, including a list of shared vocabulary.

At each store visit, we conducted two to three in-depth 45–60-min interviews with a combination of Champions, non-Champion workers, and/or Store Directors. Interviews were conducted in a private, quiet room such as an office or empty break room and were audio recorded using two devices. Topics included general worker roles, responsibilities, and chains of command; the end-to-end process of food coming into, being in, and leaving the store; the food donation program, including program rollout, goals, processes, evaluation methods, incentives, and worker feedback about the program; Company X grocery retail stores' histories, cultures, and workers' feelings about food donation; and donation partners including store relationships and donated food.

The Champions recommended that the research team participate in unstructured “interactive observations”, in which members of the research team were led around the department and/or store by a worker to observe the workers' daily tasks firsthand and hear their commentary. We found that starting visits with interactive observations built rapport and allowed more honest and transparent sharing during subsequent interviews. In a few cases, we combined in-depth interviews with interactive observations, either for convenience or because we recognized that the participant was more comfortable in this setting. In these instances, we used audio recording; otherwise, the research team took extensive notes during interactive observations, including verbatim notes when possible. We conducted interviews and/or interactive observations with all non-Champion workers who were willing to participate in the study (*n* = 30, 83%) and Store Directors from each study store (*n* = 4, 100%).

Members of the research team visited five donation partners, including at least one affiliated with each study store. At four of the five sites, the research team helped with tasks including unloading, sorting, and storing food, setting up tables, and handing out food to clients. At all five sites, we conducted semi-structured interviews with leadership and staff, during or after tasks. Interviews typically lasted 1–2 h and were not recorded, although we took detailed notes during and after visits.

The five Company X corporate leadership interviews were structured, occurred virtually over zoom with audio recording, and lasted ~60 min. Topics included goals, evaluation, incentives, and process for the food donation program, history and culture of donations in Company X grocery retail stores, factors that facilitate and hinder donations, and recruiting and maintaining relationships with donation partners.

#### 2.5.4 Champion meetings

**Figure 3:**
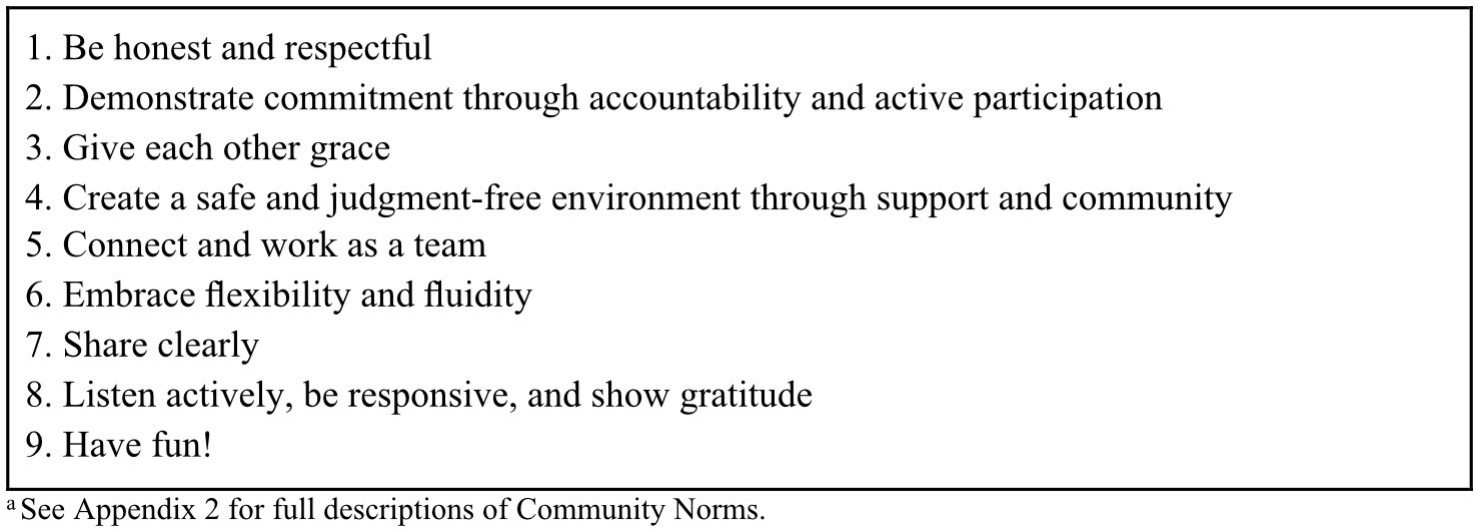
Abbreviated Community Norms created by the Champions and research team for the Food Donation Champions Project. See Appendix 2 for full descriptions of Community Norms.

We held three Champion meetings during Phase 1. All were conducted in person for 5 h at a non-company location. Food and beverages were provided. Meeting 1 (Kickoff Event) aimed to build relationships with the Champions, introduce the project, and gather initial ideas about who to interview and what to ask (Appendix 1A). We also used a participatory process to establish Community Norms, defined as ground rules, principles, and/or commitments that determine how we want to work together, as a guide to our collaboration. To do this, we asked the Champions to brainstorm processes and behaviors that they felt were important when working in teams. Through facilitated discussion, we collaboratively created nine Community Norms (Figure 3) and descriptions of each norm (Appendix 2). We reviewed the Community Norms at the beginning of each meeting for the duration of the project.

At Meeting 2 (Mid-Summer Check-In), we shared and discussed initial findings with the Champions and asked for additional suggestions on what research to conduct in the latter half of Phase 1 (Appendix 1B). The Champions recommended speaking with and drafted interview questions for subregional, regional, and national-level leaders, and requested more information about their stores' donation partners. We collectively created a list of topics for inquiry with these partners, including organization mission, number of individuals served, and the process for handling, storing, and distributing donated food.

At Meeting 3 (Synthesis), we presented what we learned from donation partners, shared and discussed additional findings, and used a collaborative process to begin drafting “insights” (summaries of phenomena, see below) based on themes and patterns found in the data (Appendix 1C).

#### 2.5.5 Data analysis

After each store visit, research team members uploaded their audio recordings to a shared location and added notes into a collaborative memo within 48 h of the visit. Memos included observations, quotes, reflections, and initial analyses regarding the visit, including relevant similarities or contrasts with data collected during other store visits or Champion meetings. We transcribed audio recordings using a professional transcription service, de-identified them, and checked them for accuracy by simultaneously listening to the recording and reading through the transcription, correcting errors as needed.

We used a convergent approach to analyze the data, drawing on qualitative methods from public health, anthropology, and design. Similar to the process for data collection, each researcher shared the process they use to analyze qualitative data, and we compared and contrasted the processes and discussed options for combining them. We created a standard operating procedure document with shared vocabulary and the steps we would use to analyze the data. In some cases, methods were similar but the design researchers described them using different words (e.g., “categorizing” vs. “coding”, “sub-categories” vs. “themes”). In other cases, methods were unique to each discipline (e.g., inductive and deductive analyses were unique to public health and anthropology (28), whereas creating insights was unique to design (22). Overall, we found many similarities between the methods used in anthropology and public health. Likewise, most data collection methods in design research are grounded in ethnographic research methods of anthropology. However, we found that design research departs from the other disciplines in its approach to testing and implementing ideas emerging from research, while anthropology and public health have a stronger commitment to impact evaluation and articulating findings for peer-reviewed publishing.

Ultimately, we used deductive and inductive methods to conduct a thematic analysis of data collected during interviews, interactive observations, and donation partner visits. We developed an initial codebook of relevant codes based on the in-depth interview guides, which we used to delineate the data from the interviews and field notes. We used Google Docs to code de-identified transcripts instead of traditional qualitative analysis software because it was familiar to all researchers and was free of cost. Using the Comments tool in Google Docs, three researchers initially coded three interview transcripts to test the initial codebook and inductively added codes. There were 13 codes in the final codebook (Appendix 3). Next, each of the remaining interview transcripts and the field notes were coded twice by six members of the research team. Each coded data point (i.e., a quote or section of text assigned to a code) was moved into a Google Sheet organized in columns bearing the codebook themes. All data points, which consisted of quotes or sections of conversations from interviews and observations from store visits, with unique identifiers for interviewees, were thus grouped by code. We generated over 650 individual data points.

We used the Mural^®^ visual collaboration platform (https://www.mural.co/) to collectively analyze the data. Mural^®^ is a cloud-based collaboration application that, in the case of this study, served as a virtual whiteboard to arrange codes, themes, and subthemes. Mural^®^ and other visual collaborative platforms are commonly used in design research but are less commonly used in anthropology and public health. We chose this platform because it emulated the tactile process of using sticky notes, allowing us to easily move individual data points, quotes, codes, and themes around during discussions and when working remotely.

**Figure 4:**
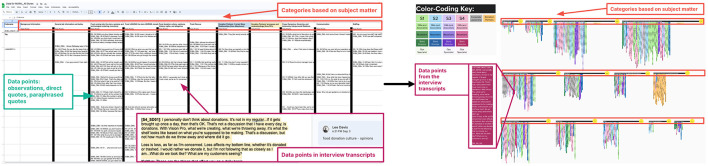
Data analysis: moving each data point from the Google Sheet to Mural^®^.

**Figure 5:**
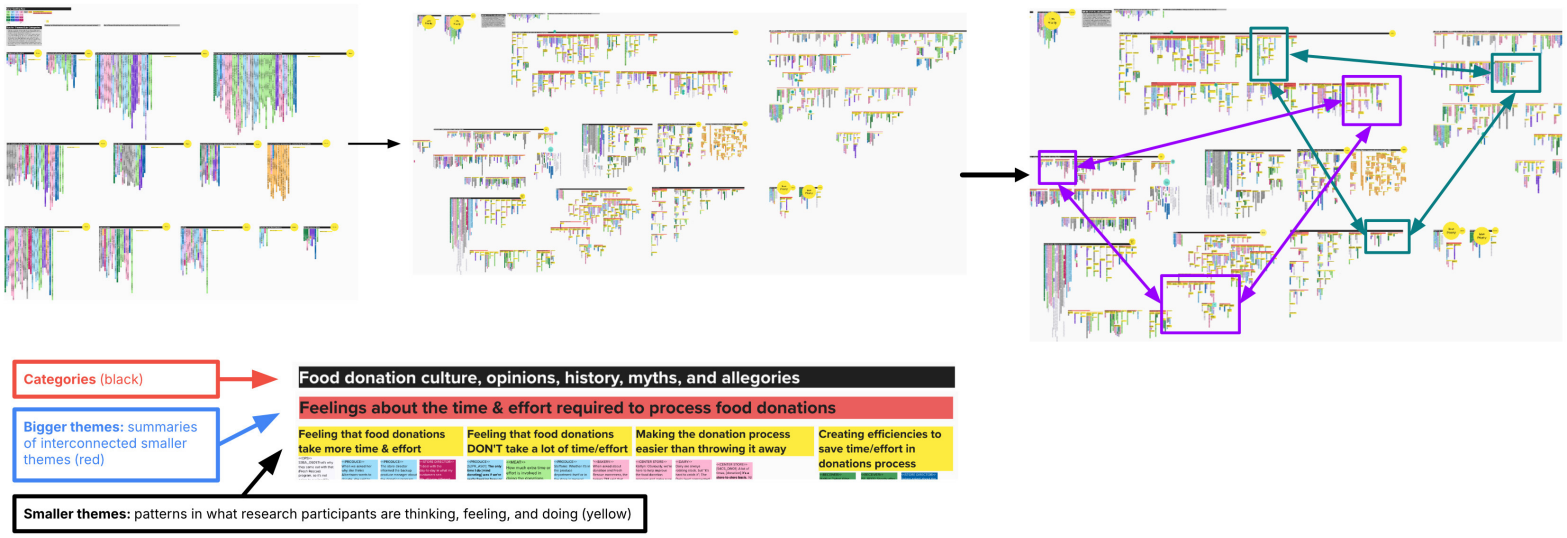
Data analysis: generating synthesized themes.

In Mural^®^, we set up the virtual whiteboard by creating separate sections for each code and moving each data point from the Google Sheet to the corresponding section on the board (Figure 4). Each data point had been tagged with the research participant's unique identifier alias and was color-coded according to their role and store. Then, each member of the research team was assigned two to four codes to categorize into smaller themes which were then aggregated under larger umbrella themes within the initial codes (Figure 5). After all data points had been assigned at least one theme, we compared and contrasted umbrella themes across codes, looking for patterns of shared or contrasting ideas, combining duplicative themes together and moving similar themes in closer proximity to one another on the whiteboard. In the end, we generated 22 synthesized themes.

At the third in-person meeting with the Champions we conducted “member checking”: presenting the synthesized themes encompassing the entirety of the data collected and exploring the extent to which these themes resonated with their experiences (29). The Champions discussed the themes in small groups with one member of the research team leading each group. They were encouraged to challenge, correct, or confirm the themes to ensure accuracy.

**Figure 6:**
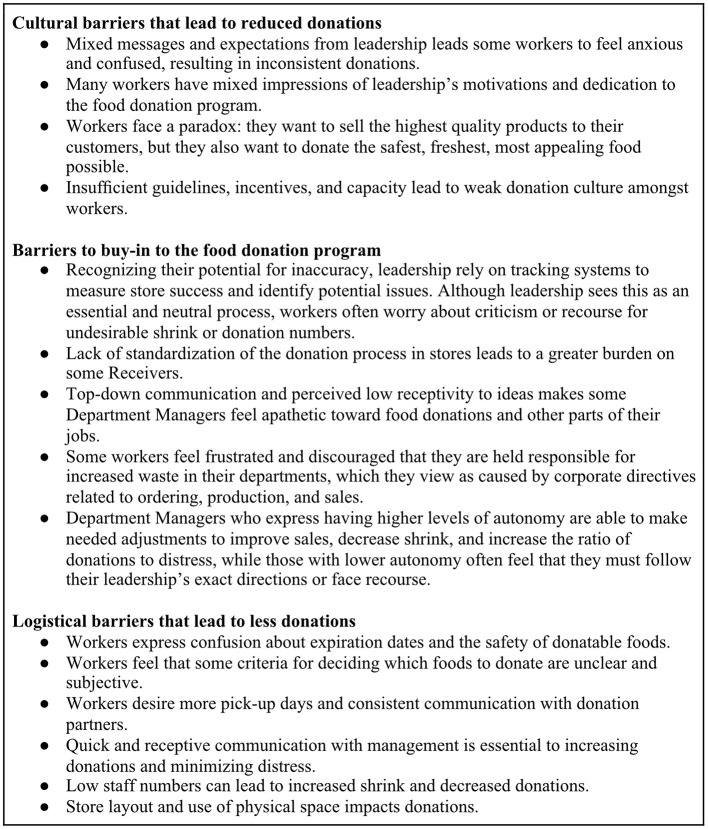
Titles of insights created by the research team based on Phase 1 data from the Food Donation Champions Project.

The final stage of synthesis involved writing “insights.” In HCD, an insight is a statement that describes a specific phenomenon identified in the data, tying multiple themes together (22). Insights are used to highlight existing tensions, conflicts, or problems, rather than, as in the term's colloquial usage, being used for any new understanding based on the data. Insights are used during the following step of the design process, ideation, to generate ideas for solutions. Insight writing is iterative and reflexive. We began the process during the third in-person meeting by teaching the Champions about insights and the insight-writing process and then writing the first two insights with the Champions. The research team met three more times to identify additional insights and finalize the insights created by the Champions. Although the process of writing insights is unique to HCD, researchers brought perspectives rooted in their respective disciplines. For example, the public health researchers used health equity and environmental justice as the basis for analysis, the design researchers focused on human behaviors, experiences, feelings, and actions, and how they complimented or were in tension with one another, and the anthropologist used social equity, structural power dynamics, and cultural conditions as lenses through which to analyze the data. In the end, we identified fifteen insights based on the data that drove ideation, the next step of the process (Figure 6). All insights reflect data specifically collected from the four participating stores and their donation partners, though the challenges identified here may also be applicable to other low donation stores across other grocery retail chains. Insights and the final strategies that were subsequently developed will be described in more detail in a forthcoming paper.

### 2.6 Phase 2: creating and testing

#### 2.6.1 Ideation

**Figure 7:**
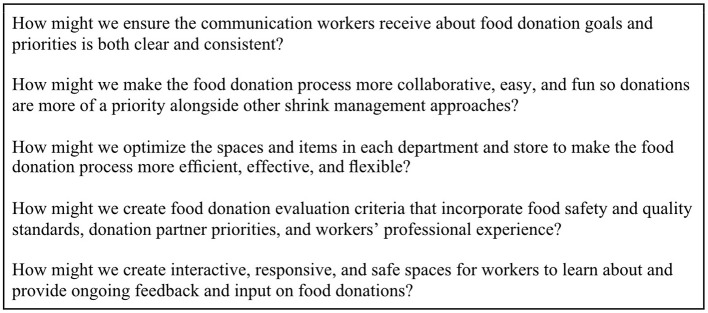
“How Might We…” opportunity questions created from research insights.

Next, we selected insights to transform into five “How Might We...?” (HMW) opportunity questions (Figure 7). HMW questions reframe insights and allow us to identify opportunities for potential interventions (22). Each question does not necessarily align with a single insight but rather draws from ideas observed in multiple insights. Due to limited time and capacity, we chose to only transform the insights that could be addressed through strategic store-level interventions rather than those that would be better addressed through system-wide changes in the company.

During the fourth in-person meeting, we brainstormed ideas to address the HMW questions with the Champions (Appendix 1D). We encouraged the Champions to generate as many ideas as possible, prioritizing quantity over quality. Brainstorming activities aimed at eliciting novel and creative ideas included (1) thumbnail sketching; i.e., posing HMW questions and having workers draw pictures of as many ideas for solutions as possible in a two-minute period (30); (2) alternative worlds; i.e., asking the Champions to imagine how they would create solutions if they were addressing the HMW questions through the perspectives of well-known companies, celebrities, characters (31); and (3) creative matrices; providing situational prompts (e.g., What if we had unlimited resources? What if we were in the year 3023?) (32). Through the ideation process, the Champions and research team generated over 400 ideas. Ideas were then quickly synthesized by the research team and the Champions voted using sticker dots to prioritize five ideas to be further developed in the next step (33).

#### 2.6.2 Prototype development and testing

At the fifth in-person meeting with the Champions, we began developing prototypes of each of the prioritized ideas (Appendix 1E). In design research, a prototype is a model or activity created to test the desirability, usability, and/or feasibility of a concept or idea (22). Prototypes vary based on the details or features being tested and may not look like the final product. Throughout development and testing, prototypes range from low to high fidelity, according to how far along they are in the development and testing process and how close the final prototype is to being ready for implementation. At the meeting, the Champions fleshed out the details (who, what, when, and where) and potential features of each of the five ideas. The Champions worked in small groups and moved through stations to provide feedback on each of the five ideas using activities such as storyboarding (visually drawing and writing out how an event will play out, scene by scene) (34), card sorting (a research method in which study participants place individually labeled cards into groups according to criteria that make the most sense to them) (35), and sticky note brainstorming (similar to card sorting but participants develop each idea on sticky notes prior to sorting) (36). When planning this meeting, we found that these hands-on activities are commonly used in both HCD and public health (commonly used in formative research and CBPR).

After the meeting, the research team summarized the Champions' input and created low fidelity versions of each prototype. The research team met three times as a whole group and multiple times in smaller groups to discuss and create the initial prototypes. After careful consideration, we decided that four of the five ideas created by the Champions were suitable for prototyping and one idea was better suited as a recommendation. We created visual representations of each of the four prototypes to share with the Champions during prototype testing. The visuals included service blueprints (charts used to visually map out the steps in a service process) (37), rough drafts of posters, and a written outline of a training curriculum. Additionally, we created a list of interview questions that focused on evaluating the desirability, usability, and feasibility of each prototype.

The research team visited each of the study stores three times during prototype testing. At each store visit, we met with the store's Champions for 60–90 min to obtain feedback. These interviews were semi-structured and documented through written notes. After each round of in-store meetings, the research team revised the prototypes based on Champion feedback. Given the timeframe of the project, we were able to develop two high- and two medium-fidelity prototypes, as well as a brief narrative for a non-prototype recommendation.

#### 2.6.3 Final share back with corporate leadership

**Figure 8:**
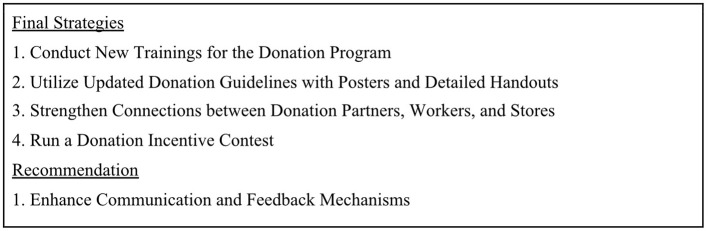
Titles of four final strategies and one recommendation to reduce wasted food and improve food donation created during the Food Donation Champions Project.

At the final in-person meeting, the research team and the Champions co-presented the final prototypes to five members of corporate leadership, including national-level directors (Appendix 1F). Upon recommendation from our corporate partners, we used the term “strategies” to describe the final prototypes (Figure 8). These strategies will be described in detail in a future manuscript. Each strategy was presented by a member of the research team and two Champions. Additionally, we provided visual representations of each strategy for corporate leadership to review.

In the first half of the meeting, we met with the Champions without leadership to discuss results and practice their presentations. We also presented the Champions with superlative awards (e.g., Brightest Smile Award, Early Bird Award) and Certificates of Achievement for completing the project. Our priority was to ensure the Champions felt appreciated, excited, and comfortable sharing our collective findings with leadership.

#### 2.6.4 Exit interviews

In the month following the Final Share Back, we conducted exit interviews with each Champion. Interviews were conducted in each of the Champions' respective stores and lasted 60–120 min. The interviews focused on overall reflections about the Food Donation Champions Project and included topics such as what they liked about the project; what they would have changed; how their perceptions changed before and after the project with regards to corporate leadership, themselves and their roles as workers, and food donation; and final reflections about each of the four strategies.

## 3 Discussion

In this paper, we describe an innovative, convergent, and worker-centered approach by public health, anthropology, and design researchers and grocery retail frontline workers to co-create interventions aimed at reducing wasted food and improving food donation. Below, we describe three key lessons learned from the project.

### 3.1 Worker-centered projects require creating an environment of collaboration and trust to enable workers to contribute with honesty

The research process and resulting strategies presented here would not have been possible without the expertise of the Champions at every stage. We noted three ways in which they engaged in the project. They participated in every in-person meeting, despite having to travel, in some cases, over an hour from their stores and having other personal and professional responsibilities and obligations. They were willing to share their experiences openly and genuinely throughout the research process, while offering expert advice on store operations, food handling, employee relations, and more. And they acted as liaisons between us and other workers in their stores, sharing their learnings after each meeting and stepping up to be true food donation champions in their stores without prompting. But what factors led to such deep engagement by the Champions?

First, we want to acknowledge that the workers who chose or were chosen by leadership to participate in the project were already outstanding leaders; each individual brought a wealth of knowledge and expertise stemming from years, if not decades, of experience in grocery retail. All Champions were invested in the company's success and dedicated to bettering themselves, their coworkers, and the community. Yet, as in many large businesses, despite their capability and dedication, these workers described limited opportunities to meaningfully contribute to key decisions in their stores. Many felt their ideas were not valued by leadership and/or did not trust that leadership always had their best interests in mind. Over time, this led to workers feeling discouraged from sharing feedback about systems and processes that they felt were inefficient, ineffective, and/or wasteful. These sentiments became a central theme during the first in-person meeting and throughout Phase 1 of the project.

Many businesses describe their efforts as worker-engaged or as including workers, but in our experience, there is a significant difference in both process and outcome between “worker-engaged” and “worker-centered” approaches. The former tend to include “informing,” “consulting” and at best, “involving” approaches [based on a continuum published by Colorado State University (38)], while our worker-centered approach focused on “co-creation.” We treated the Champions as equal partners throughout the project by asking for their opinions, actively listening to their responses, and incorporating their feedback into the research process. The research team also sought to create a welcoming environment for workers, where they could share their opinions and know they were heard and that their confidence would be kept. We prioritized getting to know the Champions personally and professionally through icebreaker activities and games at the beginning of each in-person meeting. We paid attention to small details, such as providing the Champions' preferred foods at in-person meetings and celebrating birthdays and other special occasions with cards and sweets. The Champions also reported that the atmosphere and experience were “fun,” which enabled them to enjoy the experience, participate more fully, and look forward to returning for each meeting.

Additionally, we communicated project updates on a biweekly basis by texting, calling, or emailing the Champions, keeping them invested in the project over the 10-month study period. These actions produced a relationship of reciprocity and led to the co-creation of uniquely tailored results and interventions that would not have been possible without the deep collaboration between the Champions and researchers throughout the project. When we asked them why they trusted us in the exit interviews, they noted that we stayed true to our commitment of including them in decision making, treated them as equals, and incorporated their ideas throughout the project. Many Champions also mentioned that it was impactful that we co-created a shared list of Community Norms at the beginning of the project and, importantly, that we reiterated those norms every time we met. Notably, nearly all of the Champions stated in their interviews that they did not inherently trust us, but rather, that trust was built over time through consistency and alignment of our stated intentions with our actions. The Champions also noted that they trusted us more than they otherwise would have because, although we collaborated with members of corporate leadership, we were not affiliated with the company. This provided a unique opportunity for workers to be honest without concern of recourse.

Although businesses often state the aim to incorporate worker engagement or feedback in the development of some of their initiatives, there is still room for progress. We recognize that not all grocery retail stores have partnerships with research institutions or the resources to hire external facilitators, and further, that the Champions in this project were only ten of the thousands of workers employed by Company X. However, much of the essence and approach that we modeled in this methodology can also be incorporated into company practices by corporate leadership to engender more authentic worker-centered involvement. Notably, it may take extra time and effort to build trust with workers in corporate-led initiatives, as they may be used to the standard hierarchical structure and culture of top-down decision making. Over time, by seeing their ideas turn into concrete solutions, workers may feel more comfortable and willing to share with leadership.

The Champions recommended that leadership prioritize interactive communication and feedback to help workers feel secure and welcome in sharing input. Improving communication and feedback loops in the food donation process would enable grocery retail companies to better learn from and leverage the expertise of their extensive network of frontline workers. Additionally, we strongly recommend that any grocery retailers interested in implementing these strategies center frontline workers' input, perspectives, opinions, and ongoing feedback. Engaging workers in the process of implementation has been shown in numerous studies to improve intervention success, including the likelihood that the strategies will reach, resonate with, and motivate retail workers, increasing the overall effectiveness of the strategies in reducing waste and improving food donation (39). A meta-analysis of research on work engagement also finds overall positive associations with worker performance and reduced absenteeism (40).

### 3.2 Worker-centered research requires blending and balancing the roles of researchers, facilitators, consultants, and advocates

In non-participatory qualitative research projects, participants provide information and researchers synthesize and interpret the information and make conclusions based on theories and frameworks. However, in community- and worker-centered projects such as this one, the researchers and participants' roles often blend together, and synthesis, interpretation, conclusions, and some data collection tools are co-created. In this project, the research team members played four distinct but overlapping roles: (i) researchers, who gathered and analyzed information and drew conclusions; (ii) facilitators, who taught the Champions about the research process, including how to create an interview schedule, how to analyze results, and how to synthesize ideas, and who led the Champions through this process to co-create strategies through engaging, in-person activities and conversations; (iii) partners, who worked with leadership and staff to develop strategies that could benefit the company's goal of improving its environmental and economic outcomes (Note: we were not paid by the company and we emphasize that the intent of our work, as evidenced in this manuscript and other writings, is to share findings broadly rather than to benefit one company); and (iv) advocates, whose primary responsibility was to use our position of power, as faculty at distinguished universities, to encourage and defend our processes and methodologies, and furthermore, ensure the Champions' ideas and opinions were heard and prioritized by members of Company X leadership.

In some ways, these roles reinforced each other. Our engagement in the four roles led to greater investment by the research team in the process and outcomes. Our responsibility for all of these aspects of the project and for the comfort and dignity of the Champions heightened our commitment to them as individuals and experts, their ideas, their investment, their ability to participate, and their voices as agents of change in their workplaces. Further, as the Champions saw that we consistently advocated on their behalf to corporate leadership, they were more willing and excited to show up and put effort into the co-creation process. Additionally, our rigorous research methods provided credibility to our results. With this credibility, we were able to further embody our roles as advocates and push the Champions' ideas forward in meetings and communications with corporate leadership. We continually noted to leadership that the Champions contributed at every stage of the research, and that the proposed strategies would not have been possible to develop without their expertise and commitment. Leadership was receptive to the strategies and valued the Champions' expert involvement.

This study sought to challenge power dynamics from the top-down decision-making structure that typically exists in corporations. This framing impacted all aspects of the research study, from the types of questions we asked in our interviews to the epistemological framing of the thematic analysis to the verbiage used in the dissemination of results. Additionally, navigating the interplay of partnering with Company X leadership and advocating on the Champions' behalf was a challenge. There is an inherent tension between running programs that are good for communities and running a business. Company X leadership was committed to environmental sustainability, cost savings and operational efficiencies, and wanted to see workers' input incorporated into sustainability initiatives. They also played a crucial role in the logistics and operations of the project, providing access to workers and stores, facilitating communication between the research team and regional- and subregional-level leadership, helping with store recruitment, organizing store visits, and providing food, covering travel costs, and arranging logistics for the Champions to attend in-person meetings. Company X leadership also had to balance their desire for change with numerous constraints and pressures, such as those related to feasibility, cost, food safety, corporate priorities, and labor requirements. At times, the ideas we advocated for—particularly those that called for changes that affected, but were not directly related to, food donation (e.g., more labor hours to manage tasks)—conflicted with these constraints. In those situations, the research team learned to shift expectations born of conducting research in academic or non-corporate environments to accommodate the needs put forth by Company X, just as Company X accommodated our desire to include relevant data, findings, and strategies in the final report. We worked closely with Company X leadership to negotiate the verbiage of the final report so that both partners could feel satisfied with and proud of the result. In addition to these constraints, Company X leadership also felt pressure to implement changes quickly. Meanwhile, the research team had to advocate that although our process would take more time, in the long run, it would yield more responsive, effective, and lasting interventions.

### 3.3 Convergent research leads to novel processes and collaborations

While our primary goal for this project was to demonstrate a worker-centered approach to reducing retail food waste, our convergent research approach is itself an important outcome and contribution to the field as it shows great potential for further application. Convergent research can be compared to making a smoothie, in which the individual ingredients are blended together and no longer singularly identifiable to create a new flavor and texture (41). In this project, we combined the “ingredients” of HCD, anthropology, and public health approaches, along with the expertise and experiences of frontline workers and the input of corporate leadership, to create a novel and unique methodology. Each researcher brought flexibility, openness, resourcefulness, and adaptability to the project in order to expand their own repertoire of methodologies on the project and beyond.

Convergent research takes patience and humility: each member of the project, including researchers, Champions and Company X leadership, was willing to share ideas, knowledge, and resources generously; let go of predetermined methods and mindsets; and step out of their comfort zones to learn from and support one another. We each also thoroughly interrogated and assessed our own learned practices both to share them with the other members and to consider which of our own approaches could be enhanced or even rejected in favor of more effective ones learned from team members from other disciplines. Each of the groups represented contributed to the efficacy of the project and to everyone's toolkits.

Additionally, the process of convergence requires a significant time investment. The research team met weekly, sometimes for multiple hours, and held at least one all-day, in-person meeting per month for the duration of the project. The two project leads, a public health researcher and a design researcher, worked intimately together throughout the project and extensively discussed all decisions related to process and methods. The result of the time- and energy-intensive process of convergence was both rewarding for the research team and valuable toward the project goals. Our team and methodology was strengthened, yielding more thorough research and data, more creative ideas, and more in-depth relationships with the Champions, Company X leadership, and one another.

Our research team is committed to continue evolving and testing this unique approach and is already collaborating to identify further opportunities to apply this convergent, worker-centered research method to other wasted food challenges and toward addressing other critical social and environmental challenges.

### 3.4 Limitations

Despite the many strengths highlighted in this paper, this project had some limitations. Company X has multiple regions across the US. Our research was conducted in one region with four stores that were either not donating or donating very little on a regular basis prior to the beginning of the study. Although qualitative research does not require the same number of participants as quantitative research for the findings to be rigorous, the results of this study may not be generalizable to all Company X stores. That said, in HCD and similar methodologies, it is often best practice to design for the extremes. This ensures what is designed is inclusive and accessible, and that all users generally benefit. With that principle in mind, although these strategies may make the most impact in stores that are not donating or are donating a limited amount, the approach can improve engagement and effectiveness at all grocery retail stores. We note that the purpose of this study was to design the strategies, but future research is needed to determine the effectiveness and understand the best ways to implement them. Finally, although we interviewed numerous key personnel throughout the study, including Champions, non-Champions, members of corporate leadership, and food donation partners, we were not able to interview individuals from all parts of grocery retail (e.g., distributors, waste haulers, customers). Future research may consider involving such individuals to identify additional opportunities and barriers to reducing food waste and improving food donation.

### 3.5 Conclusion

Worker-*engaged* projects are relatively common, but few projects invest time in building trust and truly *centering* the views of those most closely involved in the day-to-day tasks connected to a project's goals. We offer the approach here to provide guidance on how we implemented the Food Donation Champions project and to share features of the work that contributed to its effectiveness. At the same time, by highlighting the benefits of convergence, part of the lesson is encouragement to other researchers to “make your own smoothie.” Fresh blends of expertise from researchers, workers, and business leadership can only strengthen the effectiveness of projects that elevate the lived experience and expertise of the people most central to addressing social and environmental problems.

## Data Availability

The datasets presented in this article are not readily available because these data contain proprietary information that may reveal the identity of the industry partner and may not be shared. Requests to access the datasets should be directed to kharpe14@jhu.edu.
