# Supplementary material for: Methods for using worker-centered research to improve food donation and reduce wasted food in a grocery retail setting
Source: Front Public Health. 2025 Jul 18;13:1609717. doi: 10.3389/fpubh.2025.1609717 (PMC12313571; doi:10.3389/fpubh.2025.1609717)
Supplement: Supplementary file 1 [file Table_1.docx]

**APPENDICES**

**Appendix 1: Goals and Agendas for Each Champion Meeting**

1. Kickoff Event
2. Mid-Summer Check-In
3. Synthesis Meeting
4. Ideation Meeting
5. Prototyping Meeting
6. Final Share Back Meeting

**Appendix 2: Community Norms**

**Appendix 3: Analytic Data Codes and Descriptions**

**Appendix 1: Goals and Agendas for Each Champion Meeting**

*Appendix 1A: Kickoff Event*

**Goals**

By the end of the day, you will be able to…

1. Know the names of everyone involved in the project (Champions and Researchers).
2. Explain what the Food Donation Champions Project is and describe your role in the project.
3. Describe the key causes and impacts of food waste, and name some ways to reduce food waste.
4. Conduct a short Food Waste Conversation with a peer using research techniques.

**Agenda**

| 9:30 AM - 9:45 AM | Check in, coffee / tea / snacks |
| --- | --- |
| 9:45 AM - 9:50 AM | Welcome |
| 9:50 AM - 10:15 AM | Introductions + team building activity |
| 10:15 AM - 11:00 AM | Project overview and logistics |
| 11 AM - 11:15 AM | Break |
| 11:15 AM - 12:15 PM | Establishing community norms activity |
| 12:15 PM - 12:45 PM | Lunch |
| 12:45 PM - 1:15 PM | Wasted Food 101 |
| 1:15 PM - 2:00 PM | Research tools and techniques |
| 2 PM - 2:10 PM | Break |
| 2:10 PM - 2:30 PM | Next Steps and Q&A |

*Appendix 1B: Mid-Summer Check-In*

**Goals**

By the end of the day, we hope you will…

1. Be reminded of the project purpose, process, roles, (updated) timeline
2. Share experiences about how it has been to be a Champion
3. Provide input to researchers for the final few weeks of data collection
4. Have fun!

**Agenda**

| 9:15 AM - 9:45 AM | Check in, coffee / tea / snacks |
| --- | --- |
| 9:45 AM - 9:50 AM | Welcome |
| 9:50 AM - 10:10 AM | Introductions + Champions Circle activity |
| 10:10 AM - 10:25 AM | Review project goals and logistics |
| 10:25 AM - 11:05 AM | Champions Check-in |
| 11:05 AM - 11:15 AM | Break |
| 11:15 AM - 12:15 PM | Sharing preliminary results and gaps |
| 12:15 PM - 1:00 PM | Lunch |
| 1:00 PM - 2:20 PM | Small Groups (in rotation, 40 minutes each round):   1. Decision-making mapping 2. Donation partner discussion |
| 2:20 PM - 2:30 PM | Wrap up, next steps, and Q&A |

*Appendix 1C: Synthesis Meeting*

**Goals**

By the end of the day, we hope you will…

1. Understand what we’ve done so far (data collection and organization)
2. Help synthesize the data by drafting insights about the data collected
3. Prepare for the next phase of the project by creating a set of guidelines to use in the ideation process
4. Have fun! :)

**Agenda**

| 9:00 AM - 9:35 AM | Check in, coffee / tea / snacks |
| --- | --- |
| 9:35 AM - 9:40 AM | Welcome: Today’s Goals and Agenda |
| 9:40 AM - 9:55 AM | Warm-Up: Champions Circle Part 3 |
| 9:55 AM - 10:05 AM | Review Community Norms |
| 10:05 AM - 10:20 AM | Sharing experiences from donation partner visits |
| 10:20 AM - 10:45 AM | What have we done so far? (Overview of the synthesis process) |
| 10:45 AM - 10:55 AM | Break |
| 10:55 AM - 12:00 PM | Drafting insights together - Part 1 |
| 12:00 PM - 12:40 PM | Lunch |
| 12:40 PM - 1:15 PM | Drafting insights together - Part 2 |
| 1:15 PM - 2:25 PM | Creating Design Principles |
| 2:25 PM - 2:30 PM | Conclusion and Next Steps |

*Appendix 1D: Ideation Meeting*

**Goals**

By the end of the day, we hope you will…

1. Develop a set of guidelines to use throughout the ideation process
2. Describe some of the basic findings from the data
3. Brainstorm MANY ideas to improve the food donation program
4. Begin prioritizing 3-5 ideas we want to prototype
5. Have fun! :)

**Agenda**

| 9:00 AM - 9:30 AM | Check in, coffee / tea / snacks |
| --- | --- |
| 9:30 AM - 9:45 AM | Welcome and Icebreaker |
| 9:45 AM - 10:30 AM | Creating Design Principles |
| 10:30 AM - 10:35 AM | Break |
| 10:35 AM - 12:00 PM | Introduction to Ideation and Ideation Activities |
| 12:00 PM - 12:40 PM | Lunch |
| 12:40 PM - 2:20 PM | Prioritizing Ideas (with a break!) |
| 2:20 PM - 2:30 PM | Conclusion and Next Steps |

*Appendix 1E: Prototyping Meeting*

**Goals**

By the end of the day, we hope you will…

1. Explain what prototyping is and how prototypes can differ depending on the idea
2. Participate in the first step of prototyping—fleshing out ideas
3. Provide insight on key feasibility considerations for each prototype

**Agenda**

| 9:00 AM - 9:30 AM | Check in, coffee / tea / snacks |
| --- | --- |
| 9:30 AM - 9:45 AM | Welcome and Icebreaker |
| 9:45 AM - 10:05 AM | Introduction to prototyping + Reviewing our ideas |
| 10:05 AM - 12:00 PM | Prototyping Stations (with breaks!) |
| 12:00 PM - 12:40 PM | Lunch |
| 12:40 PM - 1:05 PM | Finish Prototyping Stations |
| 1:05 PM - 2:25 PM | Key Considerations Brainstorming Activity |
| 2:25 PM - 2:30 PM | Conclusion and Next Steps |

*Appendix 1F: Final Share Back Meeting*

**Goals (Champions only)**

1. Review how far we’ve come
2. Share the final versions of each strategy for improving food donation
3. Prepare for Part 2, including Champion opportunities to present *(optional but encouraged)*
4. CELEBRATE!

**Agenda**

| 9:00 AM - 9:30 AM | Check in, coffee / tea / snacks |
| --- | --- |
| 9:30 AM - 9:40 AM | Welcome and Icebreaker |
| 9:40 AM - 10:00 AM | Superlatives and Photo Slideshow |
| 10:00 AM - 10:20 AM | Prototyping Share Back |
| 10:20 AM - 10:35 AM | Break (and Champion Birthday Surprise!) |
| 10:35 AM - 11:05 AM | Befores/Afters - Gathering Champion Feedback about the Project |
| 11:05 AM - 11:55 AM | Preparing to Share with Leadership |
| 11:55 AM - 12:00 PM | Closing Activity |

**Goals (Champions and Corporate Leadership)**

1. Share the final versions of each strategy for improving food donation
2. Share Champions’ reflections about the project

**Agenda (Champions and Corporate Leadership)**

| 12:00 PM - 12:30 PM | Lunch, Welcome, Norms, and Icebreaker |
| --- | --- |
| 12:30 PM - 12:45 PM | Project Overview |
| 12:45 PM - 1:45 PM | Strategies Share Back by Researchers and Champions |
| 1:45 PM - 2:00 PM | Champions Reflections Share Back |
| 2:00 PM - 2:15 PM | Presentation of Champion Certificates of Achievement |

**Goals (Corporate Leadership Only)**

1. Discuss each prototype in depth and provide an opportunity for questions and feedback
2. Discuss next steps for collaboration

**Agenda (Corporate Leadership Only)**

| 2:15 PM - 2:30 PM | Break |
| --- | --- |
| 2:30 PM - 2:45 PM | Artifact Walk |
| 2:45 PM - 4:00 PM | Prototypes Discussion |
| 4:00 PM - 4:25 PM | Discuss Next Steps for Collaboration |
| 4:25 PM - 4:30 PM | Gratitude and Closing |

#

# **Appendix 2: Community Norms**

# 1. **Be honest and respectful.** Honesty and respect creates trust. There is no trust without respect and there is no respect without honesty.

# 2. **Demonstrate commitment through accountability and active participation.** Trust that your team members will follow through on what they say.

# 3. **Give each other grace.** Although we are a team, we are also individuals with lives and responsibilities outside of this project. Be understanding and show one another empathy. Communicate with your team if you need help or if you will be unable to fulfill your project responsibilities.

# 4. **Create a safe and judgment-free environment through support and community.** Building personal connections will make us a stronger team. Knowing your teammates have your back and having opportunities to try new things allows us all to grow. Team members should have space to be themselves, share their unique experiences, and participate in their own ways.

5. **Connect and work as a team.** Even though we come from different stores, organizations, and backgrounds, we are one team. Build on the ideas of others, collaborate, and work together, not against each other.

6. **Embrace flexibility and fluidity.** Things will not always go as planned; recognizing the constraints we are all working within, be open and willing to go in different directions.

7. **Share clearly.** Communication is a two-way street. When sharing, speak your truth. Be transparent and explain the why behind your decisions or thoughts. While we should step up to share our own ideas, we also need to step back to allow others to share theirs, allowing those who are quieter or more reserved to share if and when they feel comfortable.

8. **Listen actively, be responsive, and show gratitude.** When listening, focus on what others are saying and ask for clarification if you need it. Allow your teammates to feel heard by showing them you are listening. Respond to let your teammates know you’ve received their message. Show your appreciation and recognize your teammates for their contributions.

9. **Have fun!** Positive mindsets lead to increased morale, creativity, and engagement.

#

#

# **Appendix 3: Analytic Data Codes and Descriptions**

**Background information**

- Years or service or other pertinent information that is relevant to this project.

**General job information and duties**

- Daily tasks, responsibilities, role descriptions, etc.

**Food leaving the store (shrink, donations process)**

- The step by step process or "the how" of how employees are evaluating food to be distressed or donated; protocols, storage, physical space. Who does what. Includes information about physical space. Barriers to food donation. Also includes distress, compost, reclamation, and theft. Also includes ideas around shrink-busting and recovery programs.
- Training other staff on the donation process

**Food coming into the store, systems and technologies**

- Information about metrics, technology for ordering, inventory, etc. Opinions about the systems and variations in use of the systems. Includes ordering and warehouse processes. Also includes information about planograms/firms/etc.

**Donation partners: Current Store Relationships (from Company X)**

- From the perspective of the stores: Who they are, how they work, people's perceptions of donation partners, relationships between stores and donation partners. More factual processes and information (does not include history of donation partners)

**Donation partners' processes and understandings (from DPs)**

- From the perspective of the donation partners; information from partner visits.

**Food donation culture, opinions, history, myths, and allegories**

- History of donation at the store and how it has worked/fluctuated over time. Interpretations of food donation and partners. Also includes information about tax incentives around donating

**Food Donation Program**

- Information about the food donation program rollout, thoughts/opinions on the program overall, the program policies and goals; Might be some overlap between food donation history and this category. Misperceptions about the food donation program. Includes training specifically about the program.

**Communication**

- Any communication between employees; all aspects of communication within the Company X system. Physical objects (e.g., cell phones) or manifestations of communication (e.g., signage, bulletins). Mixed messages around shrink.

**Power Dynamics, Hierarchy, and Autonomy, Interpersonal Relations**

- Information about decision-making power, who people answer to, who they manage, who they ask questions to, etc. Perceptions of corporate.
- Non-donation related incentives, like for Department Managers or Store Directors doing their jobs well.
- Also if one manager talks down about another manager

**Staffing**

- Information about staffing capacity, understaffing, budget for staffing, staff limitations, turnover; also talks about workers being too busy/pressed for time
- Training staff for day-to-day tasks (not related to donation process -- if it was related to donation, it’d be in donation process)

**Physical Space (Non-Donation)**

- Any descriptions or observations of physical space in the store that doesn't have to do with donations

**Misc. or Other**

- Anything that does not fit in the categories above
- Include here: feedback from store directors about their store’s involvement/their involvement in the research project

# 
